# Supplementary material for: Reports on sexual violence published in an online Chinese newspaper: A new frame research
Source: PLoS One. 2024 May 10;19(5):e0299578. doi: 10.1371/journal.pone.0299578 (PMC11086924; doi:10.1371/journal.pone.0299578)
Supplement: Attachment — Submitted filename: PONE-D-23-12920_reviewer.pdf [file pone.0299578.s001.pdf]

This is a documentary research on the news of sexual violence disclosed in the People's Daily Online. Its theme is current, relevant and brings reflections on how the media can influence society in a positive or negative way, depending on the content, format and the focus of the published reports.
However, the material lacks critical review and restructuring, especially with regard to the sections "Materials and Methods", "Dissucsion" and "Conclusion". subtitle formatting.

A detailed evaluation of the manuscript follows, with suggestions and recommendations to help authors improve the material.

Title: I propose the following essay “Reports on sexual violence published in an online Chinese newspaper: a new frame research”

Abstract:

- It does not present information related to the "materials and method" section.
- Review the wording of the excerpt "People's Daily Online, a representative online media in China, was taken as the research object, to analyze the sexual crime reports in the past 15 years.”, in order to make the objective clear to the reader of the article.
- Pay attention to the time frame mentioned, which is not the same as mentioned in the text of the manuscript, where the authors report 20 years.

Introduction: Does not present the purpose of the manuscript at the end of the section. As a suggested objective, I propose: To describe the characteristics of the news about sexual violence published in the People's Daily Online.

Theoretical Background:

- The title level of subsections is not presented according to publication standards.
- Note that, in some paragraphs, there are excerpts with wording that resemble the objectives of the study
- Review the last paragraph of this section, as its content has multiple objectives, which is not appropriate for a scientific article.
- Research Questions: This is not a section of the manuscript in accordance with the journal's guidelines.

- I suggest including the content presented in item 3 in the introduction, making the necessary adjustments in the wording.
- Regarding the guiding questions of the research, I recommend the presentation of only one guiding question, which encompasses the central idea. For example: what are the characteristics of the news about sexual violence published in People's Daily Online?

Methodology: The title of this section, according to the rules of the Journal is “Materials and Methods”. Still, it is necessary to critically review the text considering the recommendations of the COREQ checklist and the journal's rules regarding the structure of this section.

- The content presented in this section does not allow the replicability of the study. The methodological path developed by the authors is not clear.
- I recommend describing the data collection instrument applied to the reports.
- What is the type of study?
- What was the method adopted for data collection? It is necessary to cite the reference used as the basis for the methodological route.
- What version of the SPSS 19.0 program was used?
- Justify the change in the time frame considered the survey of publications. Initially the proposal was 20 years, but “from January 1, 1997, when People’s Daily Online was established, to December 31, 2019”, totals 22 years and 11 months. Next, the authors say that the cut was from October 10, 2004, to December 31, 2019”, which totals 15 years and 2 months. The reader is confused and this information conflicts with that presented in the abstract.
- When did data collection take place? who performed it? what was the instrument adopted for data collection?
- Since Python is a programming language, the text needs to be more specific about how this language was used. Through a search engine? Crowler? Which network did Python access? Python is a programming language, but was the feature adopted for research purposes? Justify the use of this language in the research.
- What were the inclusion and exclusion criteria adopted for the selection of news?
- Explain the adoption of 5% of publications for coding
- The authors present “Operational definition and analysis” as a new section of the manuscript, however, its content is part of the “Materials and Methods” section, so it must be presented as a second level of subtitle. :

- As numbered sub-items, the authors present the variables considered in the study, that is, what was extracted from the data. This information should be presented in a second subheading level called “Data collection”.
- Pay attention that, when presenting the variables (the quantity of news reports, Place of crime, the characteristics of sexual crime reports, The information source of a report, who is the persons in the news, the focus of media attention, Attribution of case responsibilities and frames focusing), sometimes the authors present data, which is not appropriate for the “Methods” section, as well as presenting information regarding the analytical process.
- From subitem 9 ("(9) Coding and reliability verification"), the authors present contents referring to a second level of subtitle of the section "Materials and Methods" called "Data analysis”.

Results: The authors present data that respond to the study's guiding questions, but the tables do not meet the Journal's training standards.

Conclusions and Discussions:

- According to the journal's rules, the section to be presented after the results is the “Discussion” section, which can be presented together with the results. However, “Conclusions” is another section, which cannot be presented together with the “Discussion” section”.
- The text in this section lacks a broad critical review, as the content consists of the authors' interpretations of the results, without any inference from the authors or dialogues with the findings of other research. This note is evident when observing that no reference was cited in this section.
- I emphasize that the text presented does not reach the depth required for an analysis, as proposed by the authors.

References:

- The list of references does not follow the format required by the journal.
- I strongly recommend updating most of the references used, since out of a total of 22 references, 16 are publications older than 5 years.
