# Supplementary material for: Reports on sexual violence published in an online Chinese newspaper: A new frame research
Source: PLoS One. 2024 May 10;19(5):e0299578. doi: 10.1371/journal.pone.0299578 (PMC11086924; doi:10.1371/journal.pone.0299578)
Supplement: Attachment — Submitted filename: Reviewed_Aug2023_PONE-D-23-12920.pdf [file pone.0299578.s003.pdf]

PLOS ONE

CHINESE SEXUAL VIOLENCE NEWS VIEWED FROM NETWORK NEWS: BASED ON NEW FRAME RESEARCH

--Manuscript Draft--

**Manuscript Number:**	PONE-D-23-12920
**Article Type:**	Research Article
**Full Title:**	CHINESE SEXUAL VIOLENCE NEWS VIEWED FROM NETWORK NEWS: BASED ON NEW FRAME RESEARCH
**Short Title:**	CHINESE SEXUAL VIOLENCE NEWS VIEWED FROM NETWORK NEWS
**Corresponding Author:**	Yang Liu<br>Xihua University<br>Chengdu, CHINA
**Keywords:**	Sexual crime report; Social service; representation of women; health guidance; sex-role stereotype
**Abstract:**	The leading role of the media is very important in the new media era in order to build the reporting system and framework of sexual violence crimes, guide the awareness and public opinion of the people, and improve the vigilance of society on sexual crimes. In this study, People's Daily Online, a representative online media in China, was taken as the research object, to analyze the sexual crime reports in the past 15 years. Research results show that, firstly, significant changes have taken place in the number of sexual crimes news reported. In the second place, those news reports on sexual crimes have the most cases in the Chinese mainland. Thirdly, the emphasis on news topics and persons is relatively concentrated on the perpetrators. Fourthly, People's Daily Online reports on sexual crimes focus the blame on the perpetrators. Fifthly, sexual crimes show that the frame is more episodic. Hoping to master how the media report social focus issues by verifying the changes in reporting of sexual crimes in China, which will become the basic information of social health, psychological cognition and guidance, and media policies in the future and provide important enlightenment for the current situation.
**Order of Authors:**	Yang Liu
**Additional Information:**	
**Question**	**Response**
**Financial Disclosure**<br><br>Enter a financial disclosure statement that describes the sources of funding for the work included in this submission. Review the submission guidelines for detailed requirements. View published research articles from *PLOS ONE* for specific examples.<br><br>This statement is required for submission and **will appear in the published article** if the submission is accepted. Please make sure it is accurate.	The author(s) received no specific funding for this work.

Powered by Editorial Manager® and ProduXion Manager® from Aries Systems Corporation

**Unfunded studies**<br>Enter: *The author(s) received no specific funding for this work.*<br><br>**Funded studies**<br>Enter a statement with the following details:<br>• Initials of the authors who received each award<br>• Grant numbers awarded to each author<br>• The full name of each funder<br>• URL of each funder website<br>• Did the sponsors or funders play any role in the study design, data collection and analysis, decision to publish, or preparation of the manuscript?<br>• **NO** - Include this sentence at the end of your statement: *The funders had no role in study design, data collection and analysis, decision to publish, or preparation of the manuscript.*<br>• **YES** - Specify the role(s) played.<br><br>* typeset	
**Competing Interests**<br><br>Use the instructions below to enter a competing interest statement for this submission. On behalf of all authors, disclose any competing interests that could be perceived to bias this work—acknowledging all financial support and any other relevant financial or non-financial competing interests.<br><br>This statement is required for submission and **will appear in the published article** if the submission is accepted. Please make sure it is accurate and that any funding sources listed in your Funding Information later in the submission form are also declared in your Financial Disclosure statement.<br><br>View published research articles from *PLOS ONE* for specific examples.	The authors have declared that no competing interests exist.

Powered by Editorial Manager® and ProduXion Manager® from Aries Systems Corporation

**NO authors have competing interests**<br><br>Enter: *The authors have declared that no competing interests exist.*<br><br>**Authors with competing interests**<br><br>Enter competing interest details beginning with this statement:<br><br>*I have read the journal's policy and the authors of this manuscript have the following competing interests: [insert competing interests here]*<br><br>* typeset	
**Ethics Statement**<br><br>Enter an ethics statement for this submission. This statement is required if the study involved:<br><br>• Human participants<br>• Human specimens or tissue<br>• Vertebrate animals or cephalopods<br>• Vertebrate embryos or tissues<br>• Field research<br><br>Write "N/A" if the submission does not require an ethics statement.<br><br>General guidance is provided below. Consult the submission guidelines for detailed instructions. **Make sure that all information entered here is included in the Methods section of the manuscript.**	N/A

Powered by Editorial Manager® and ProduXion Manager® from Aries Systems Corporation

**Format for specific study types**<br><br>**Human Subject Research (involving human participants and/or tissue)**<br>• Give the name of the institutional review board or ethics committee that approved the study<br>• Include the approval number and/or a statement indicating approval of this research<br>• Indicate the form of consent obtained (written/oral) or the reason that consent was not obtained (e.g. the data were analyzed anonymously)<br><br>**Animal Research (involving vertebrate animals, embryos or tissues)**<br>• Provide the name of the Institutional Animal Care and Use Committee (IACUC) or other relevant ethics board that reviewed the study protocol, and indicate whether they approved this research or granted a formal waiver of ethical approval<br>• Include an approval number if one was obtained<br>• If the study involved *non-human primates*, add *additional details* about animal welfare and steps taken to ameliorate suffering<br>• If anesthesia, euthanasia, or any kind of animal sacrifice is part of the study, include briefly which substances and/or methods were applied<br><br>**Field Research**<br><br>Include the following details if this study involves the collection of plant, animal, or other materials from a natural setting:<br>• Field permit number<br>• Name of the institution or relevant body that granted permission	
**Data Availability**<br><br>Authors are required to make all data underlying the findings described fully available, without restriction, and from the time of publication. PLOS allows rare exceptions to address legal and ethical concerns. See the PLOS Data Policy and FAQ for detailed information.	Yes - all data are fully available without restriction

A Data Availability Statement describing where the data can be found is required at submission. Your answers to this question constitute the Data Availability Statement and **will be published in the article**, if accepted.<br><br>**Important:** Stating 'data available on request from the author' is not sufficient. If your data are only available upon request, select 'No' for the first question and explain your exceptional situation in the text box.<br><br>Do the authors confirm that all data underlying the findings described in their manuscript are fully available without restriction?	
**Describe where the data may be found in full sentences. If you are copying our sample text, replace any instances of XXX with the appropriate details.**<br><br>• If the data are **held or will be held in a public repository**, include URLs, accession numbers or DOIs. If this information will only be available after acceptance, indicate this by ticking the box below. For example: *All XXX files are available from the XXX database (accession number(s) XXX, XXX.).*<br>• If the data are all contained **within the manuscript and/or Supporting Information files**, enter the following: *All relevant data are within the manuscript and its Supporting Information files.*<br>• If neither of these applies but you are able to provide **details of access elsewhere**, with or without limitations, please do so. For example:<br><br>*Data cannot be shared publicly because of [XXX]. Data are available from the XXX Institutional Data Access / Ethics Committee (contact via XXX) for researchers who meet the criteria for access to confidential data.*<br><br>*The data underlying the results presented in the study are available from (include the name of the third party*	All relevant data are within the manuscript and its Supporting Information files.

and contact information or URL).<br>• This text is appropriate if the data are owned by a third party and authors do not have permission to share the data.<br><br>* typeset	
Additional data availability information:	

Powered by Editorial Manager® and ProduXion Manager® from Aries Systems Corporation

Manuscript

Click here to access/download;Manuscript;Manuscript -with author details.docx

CHINESE SEXUAL VIOLENCE NEWS VIEWED FROM NETWORK NEWS: BASED ON NEW FRAME RESEARCH

Liu Yang[1]

Abstract

The leading role of the media is very important in the new media era in order to build the reporting system and framework of sexual violence crimes, guide the awareness and public opinion of the people, and improve the vigilance of society on sexual crimes. In this study, People's Daily Online, a representative online media in China, was taken as the research object, to analyze the sexual crime reports in the past 15 years. Research results show that, firstly, significant changes have taken place in the number of sexual crimes news reported. In the second place, those news reports on sexual crimes have the most cases in the Chinese mainland. Thirdly, the emphasis on news topics and persons is relatively concentrated on the perpetrators. Fourthly, People's Daily Online reports on sexual crimes focus the blame on the perpetrators. Fifthly, sexual crimes show that the frame is more episodic. Hoping to master how the media report social focus issues by verifying the changes in reporting of sexual crimes in China, which will become the basic information of social health, psychological cognition and guidance, and media policies in the future and provide important enlightenment for the current situation.

Keywords: Sexual crime report; Social service; representation of women; health guidance; sex-role stereotype

The author has declared no conflicts of interest

Ethical approval

This article does not contain any studies with human participants or animals performed by any of the authors.

[1] School of Literature and Journalism, Xihua University, Chengdu, Sichuan Province, China, Email address: amiaini1203@sina.com

1. Introduction

In October 2017, the actress and others launched the "Me Too" Campaign against, which triggered extensive discussions on sexual harassment and sexual assault in political, academic, cultural, and other fields besides Hollywood. In 2022, Netflix screened the world-shaking sexual crime of "Room N" in South Korea as a documentary and left a thought-provoking text at the end: "The crime of imitating Room N continues to happen all over the world." Sexual crimes also occur frequently in China and cause heated discussions in society. China society is no longer evading sexual crimes as it used to. But up to now, there are still many problems in the reports of sexual crimes, so it is very important to find out the relevant reported cases, summarize the reports, and objectively and fairly present the reported contents (Ru Xi Jia,2019).

Most people do not directly experience social focus issues or events, but indirectly through the media. In news reports, sexual crime news often becomes the focus of social attention, mostly because of the sex of the persons in the crime incident, the relationship between the persons in the incident, the combination of crime and sex, etc., which can stimulate readers' curiosity. Crime is not only newsworthy, it affects everyone (Ju-Hyun Hong,2018). It is the responsibility and obligation of the media to inform the audience about the crime, find out the social structure problems related to the crime, and create public opinion to prevent and eradicate the crime beforehand (Ahn Kwang-sik,1998). Thus, the positive significance of crime news to society is obvious. It is not limited to letting the public know about the existence of crime cases and strengthening the social early warning function, but to embody legal justice and guarantee judicial justice.

The focus of each era can be analyzed and inferred by understanding and mastering what

are important issues and values in a specific society. The media constitute news stories in specific ways according to the social order of the country and the interests of national stability (Jungah Ahn,2017). Because of this, the news frame is widely used in news reports, and it can emphasize and highlight the thoughts and issues that want to be conveyed to the audience when selecting a variety of topics, information sources, and reporting techniques. The text of the news report shows the editors’ different values and orientations and reflects their understanding and viewpoints, through which the reporter's intention and purpose of the report can also be understood.

The research on the news frame is still in its initial stage in China as a whole, which belongs to a relatively new research field and needs to be actively explored (Xiao Wei,2015). Therefore, it is necessary to make an in-depth study by means of mixed analysis in combination with China’s social characteristics, media characteristics, and audience characteristics while understanding the theories of Western countries (Wang Lingning,2009). China’s media circle should refer to the research at home and abroad to broaden the theoretical field of vision, especially exploring the news organization’s intention of text production through frame analysis, which is of profound significance.

2. Theoretical Background

(1) Problems and research necessity of sexual crime reporting in China

Nowadays, the media market in China is more liberal and personalized than before, and many events are revealed by the public before the media intervenes and are made public in the

form of news. However, there appeared a variety of anomie behaviors in related reports after the news was published, and people paid close attention to such issues as information disclosure, chat history, call recording, insufficient interviews in reported news, and biased reports. The anomie of sexual crime-related reports in China has occurred frequently in the past, so it is a problem that China media must pay attention to at present to reflect on the media standardization in sexual crime case reports (Feng Qunxing,2014).

The normative discussion on the news diffusion process of sexual crimes in China is far later than that in other countries, and there is a lack of scientific and convincing research in academic circles. According to the first research in this field, there are the following problems in the study of sexual crime reports in China: First, most of the research on sexual crimes in China is carried out in law, pedagogy, medicine, sociology, psychology, etc., insufficient in choosing online media to systematically construct sexual crimes reports from the perspective of frame theory. Secondly, there is little in-depth research on the types of sexual crimes in China. In most cases, it only raises questions, discusses the current situation and puts forward corresponding countermeasures through sexual crime news reports, almost with no empirical research on the causes and concrete development of the problems. Although significant academic achievements have been made in the research direction, the research scope is wide and vague. The research object is biased toward children, teenagers, celebrities, and other specific groups. The research method is relatively simple, and it always descriptively asks several questions, or abstractly selects the questions with the same content to study, without systematic and rigorous explanation for the problems in the production of crime reports. Most of the research results only focus on the management of state organs, and the research and

analysis of some attempted sexual crime reports are too subjective and unconvincing. Third, the research process of news reports on crime types lacks scientific integrity. The research on sexual crime news mostly focuses on specific news at present, without making statistical analyses on the temporal changes of sexual crime news. In addition, it is insufficient to statistically analyze and process the number of news reports related to sexual crimes with correct data, which does not attach importance to the application of research methodology and lacks necessary scientific and empirical research.

The focus should be on the statistical analysis of the history of sexual crime news reports based on the comprehensive narration of the types of sexual crime news documents. The initial form, characteristics, changes, and progress are also very important in news reports. In this study, the characteristics of China's social-related reports will be tracked and analyzed based on journalism theory, and the temporal changes of reporting forms adapted to the changes of the times will be studied. In particular, the problems in the production of crime reports should be systematically and strictly explained, which is not only to establish the norms and ethics of media coverage of sexual crimes but also to carry out relevant research. At the same time, the media need to conduct scientific and systematic research from the perspective of communication on how to construct a reporting system and frame for sexual crimes in the new media era, to guide national understanding and public opinion, and to enhance social vigilance for sexual crimes.

(2) The factors influencing the reality of sexual crimes in media reports

It is hoped that the report of sexual crime news can guide society to pay attention to it,

improve the public's alert and vigilance, and formulate relevant protection methods. But the media will use irritating language, enlarge events, and stimulate the public's curiosity and prying desire to gain public attention (Yong-kuk Chung,2012).

Lippmann (1922) believed that people understand reality through the media and that the media reconstructs reality, but there is a distance between reality and the reconstructed reality. The media give their collected event information logic in a variety of ways, including emphasizing any behavior or person, so the scale and frequency of media reporting events are very important in the issue-setting process. The reported quantity emphasizes the frequency and quantity of event reports from the perspective of media reports, while the spreading power of hot spots means how much influence hot spots have on society from the point of view of news value (Ju-Hyun Hon,2016). Therefore, it is very important to analyze the media content, because the media content analysis can not only infer those closed and invisible phenomena but also analyze and infer the audience, organization, and cultural background of specific content.

When reporting sexual crimes, the media not only reflects the pure purpose effect of attracting the attention of the audience by focusing on the news value from the perspective of the economic market but also includes the structural characteristics of the media environment, the comprehensive effects of journalists' personal feelings, the organizational culture of media companies, the competition between media companies, the production and distribution environment of digital news (Seon-A Moon, Bong-Geun Kim, Jin-Suk Kang.,2015; Sae-Eun Kim.,2017). Nevertheless, the role of the media in crime reporting is to provide relevant information about the incident, arouse the vigilance of the audience, and put forward solutions to prevent other losses. In particular, the report on crimes of sexual violence should discuss

whether the media has put forward adequate solutions with a balanced view and whether the media has fulfilled its original role.

(3) Literature review of sexual crime reports

According to previous research, 84.6% of women get information about sexual crimes through TV or newspaper reports. The formation of the media's attitudes and ideas toward the audience has had a significant impact (Seon-A Moon, Bong-Geun Kim, Jin-Suk Kang.,2015), which requires a close understanding of events to capture the reader's interest and attention. Thus, the media will dramatically organize events and describe them in detail(Greer, C.,2007). The news of sexual crimes is of high value because it contains elements that can attract readers' attention. No matter the combination of violence and sex, or the age of the victims of sex crimes, they will be highly concerned by the media (Gilchrist, K.,2010).

Foreign research on media and sexual crimes has been carried out extensively. The first part is about the problems in the reports of sexual crimes. Scholars (Sae-Eun Kim, Namhee Hong.,2019) analyzed the media reporting practices and the structural characteristics of media companies in South Korea on the coverage of the "Me Too Campaign", and concluded that various gender discrimination issues were exposed from the perspectives of the publishing, interview and reporting process, discussion process, and professional reporters reporting on women's focus issues within the enterprises. In addition, in the process of relevant reports, the competition of stop-press news in the news circulation environment centered on the portal website, the lack of understanding of the reporting policy of sexual crimes, and the contradictions in the myth of objectivist journalism have also been exposed. Secondly, the

researcher distinguishes the identity of the perpetrator and the victim in the process of analyzing and reporting the differences between them. The study found (Worthington, N.,2008) that although the victim-centered frame well represented their pain and trauma, the voice of the victims is reproduced through the radio, which not only fails to clarify the social structural problems that induce sexual crimes but triggers social debate. The media tried to convey the voice of the victims out of the sight of the perpetrators or through the practice of relying on investigative authorities. The problem, however, is that the structure of the press is still weak on the causes of victims of sexual crimes and how to deal with them (Yisook Choi, Eunjin Kim.,2019).

To sum up, several issues need attention in the extensive research on media and sexual crimes. First, the analysis of sexual crime reporting requires a fundamental, structural cause analysis and a statistically significant basis for research that can generalize the results. In particular, the research on the reports on the perpetrators and victims and media attention is of great significance (Ju-Hyun Hong,2017). Secondly, communication science should pay continuous attention to the constitution of the frame of perpetrators and victims. a more appropriate explanatory frame should be formed through the analysis and observation of actual media texts (Jung Hye Yang.,2010).

(4) The tool “frame” of news report analysis

How to understand, judging, and mastering information is very important in modern society where a large number of information emerge. Classifying, composing, and explaining the content through the frame can make people better understand the real world (Price, V &

Tewksbury, D & Powers, E,1997; Z. Pan & G. M. Kosicki,1993). The description of news reports not only reflects the simple individuals, media organizations, and phenomenal society but also reflects the social values, the reconstruction of specific realistic society, and other issues. The media not only have a cognitive impact on the public based on the number of reports but also have an important role in reporting those responsible for sexual crimes. “Attribution of the incident” means who is responsible for the incident, so it should be dealt with in an important way to clarify the responsibility of sexual crimes (Semetko, H. A., & Valkenburg, P. M.,2000). The study found that (Lu, Jian Ye, Chung, Yongkuk.,2019) media reporting events using a specific responsibility frame has the effect of inducing public awareness of the causal relationship between social focus issues. Therefore, it is very important and necessary to understand the reporting tendency of the offender and the victim as the center of sexual crime reporting.

Iyengar put forward the “thematic frame” and “episodic frame” in the form of TV news (Dong-Hoon Lee,·Won-Yong Kim,2012). The study found that the vast majority of rape-type news reports used episodic statements (Jung-Gyo Lee, Young-Nam Seo, Su-Jin Choi.,2009). The formal descriptions are not just textual differences but also have an impact on the public's understanding of news and responsibility attribution. News reports play a role in providing contextual clues when readers judge the focus and responsibility of events. The audience pursues a simple understanding of the news content, usually relying on easy-to-imagine things to understand the attribution, and seldom considering the factors in an all-around way (Jung-Gyo Lee, Young-Nam Seo, Su-Jin Choi.,2009). Therefore, it is necessary to study whether the news is carried out with thematic statements or anecdotal statements.

To sum up, this study will analyze the news reporting frame of sexual crimes to grasp the specific way in which the media constitutes the frame, changes, causes, and other issues, and to discuss in depth. Secondly, it is necessary to investigate from whose perspective the cases of sexual crimes constitute the news. The media's perspective on events and how they constitute the frame can be used to understand the process of communication between the news and the audience. Through the discussion of the research, this paper deeply narrates the news statement theory, summarizes the attribution theory which serves as the theoretical basis of responsibility attribution, and then focuses on the responsibility attribution to describe the frame theory.

3. Research Questions

One of the important fields of media research is the research on the role of media in the process of reflecting the composition of social reality content, in which content analysis is to analyze the transmitted information content, discover and verify the relationship between variables, and then infer other content of information in the diffusion process. About sexual crime reports, the media form news stories in a specific way according to the understanding and mastery of the social order of the country and the stability of the country, which is very important in analyzing how news describes and forms specific focus events. This study will analyze the reports of sexual crimes reported by Chinese media through People's Daily Online, and put forward the following research questions:

Question 1: What's the trend of sexual crimes reported by People's Daily Online?

Question 2 What are the trends in the places where sexual crimes are reported by the People’s Daily Online?

Question 3: What are the trends in the reporting forms of sexual crimes by the People’s Daily Online?

Question 4: What is the trend of news sources about sexual crimes reported in the People’s Daily Online?

Question 5: What is the amount of reports on sexual crimes that the relevant persons are celebrities in the People’s Daily Online?

Question 6: Is there any difference in media attention between the perpetrator and the victim in reports on sexual crimes by the People’s Daily Online?

Question 7: What is the composition of responsibility attribution between the perpetrator and the victim in the reports on sexual crimes by the People’s Daily Online?

Question 8: What is the proportion of thematic frame and episodic frame in reports on sexual crimes by the People’s Daily Online?

4. Methodology

(1) Research design

In this study, the author put forward and analyzed the research issues of sexual crime reports in the 20 years since People’s Daily Online was established. The analysis process is divided into four stages. The first stage was data import. After searching the related keywords of sexual crimes by Python, the time of the news, the content of the report, and the news links

were organized into Excel. The second stage was data cleaning. All reports were confirmed and only those related to sexual crimes were collated. The third stage was sampling. The collected reports were sampled by systematic sampling method. The fourth stage was to analyze statistics and explain information. SPSS 19.0 program was used for analysis.

(2) Research object

This study searched the news by four keywords such as “sexual crimes”, “rape”, “sexual violence” and “sexual assault”.

(3) Research time

For this study, the overall crawl search time was set to be from January 1, 1997, when People’s Daily Online was established, to December 31, 2019. According to the time when the searched keywords first appeared, the actual analysis time was from October 10, 2004, to December 31, 2019.

(4) Material selection

Python was used to search for keywords in this study, and a total of 80,385 pieces of news were captured. Data were cleaned according to the definitions, and specimens that did not meet the requirements of the relevant studies were screened out. Eventually, 13,897 samples were collected.

A total of 694 news on sexual crime were sampled with 5% of the 20 articles at intervals by systematic sampling. Before June 23, 2012, 87 pieces of news in the specimen had lapsed, and only 7 texts could be submitted. Therefore, the final effective analysis news was 600 pieces and 5% of the samples were coded by the encoder, and the reliability test between the codes was measured.

5. Operational definition and analysis

(1) The quantity of news reports

The quantity of reports has a close relationship with the value of news (Ju-Hyun Hong,2018). Therefore, in this study, the number of sexual crimes reported by the People's Daily Online was specifically calculated through data statistics, and the difference in reported quantity was analyzed based on time.

(2) Place of crime

According to the specific actual situation, the locations of reported cases were divided into "Chinese mainland", "Hong Kong, China, China, Macau, China, and Taiwan Province, China", "South Korea and Japan", "other Asian countries", "the United States, Europe and Australia", and "other countries (Africa, Latin America, etc.)", a total of six to be distinguished.

(3) Report form

To understand the characteristics of sexual crime reports, three items including "text", "image", and "text and image" were proposed. In addition, considering the characteristics of network media, six items including "video", "video and text", and "video, picture and article" were added.

(4) The information source of a report

The information source is an important part of the suggestive effect (Young-Jun Son, Juhyun Hong.,2018). The information sources herein were analyzed in seven categories, namely, government agencies and their employees, non-governmental social organizations,

experts and scholars, parties and their families, witnesses and relevant information providers on the spot, and unclear information sources and others.

(5) Whether the person concerned is a celebrity

According to the social stratum of the persons in the news in previous research (Ju-Hyun Hong,2017; Young-Jun Son, Juhyun Hong.,2018), they were divided into “entertainers”, “celebrities” and “ordinary people”.

(6) Media attention

In sexual crime reports, the focus of media attention is to emphasize “who” is the center of the perpetrator or the victim. For the purpose of analysis, news topics and personal information publicity features were divided into “media attention to topics”, “media attention to the exposure of people’s information” and “emphasis on people’s related content” to measure media attention.

To understand the centrality of victims or perpetrators in sexual crime reporting, the topics of the report were divided into “victim-centered”, “neutral” and “perpetrator-centered” (Young-Jun Son, Juhyun Hong.,2018). However, there might be some unclear topics in this study, so “other” was added.

The disclosure of personal information was also broken down according to the previous research which was set as “name”, “age”, “face”, “address”, “employer”, “exposure of people around” and “exposure of private life”. The measurement of the personal information index was based on the method of previous research (Young-Jun Son, Juhyun Hong.,2018). To measure the personal information of the perpetrator and the victim, a 3-point scale was used, with 1 point for “not mentioned at all”; 2 points for “mentioning to some extent” and 3 points

for “specifically mentioned”. For example, if the name and age were made public, it was classified as “specifically mentioned”; if only the name was made public, it was classified as “mentioned to a certain extent”; if a pseudonym was used or the name was not mentioned, it was classified as “not mentioned at all” to calculate the sum of these items to measure the personal information disclosure index. The larger the value of the personal information index is, the more detailed and specific reports would be made on the personal information of the perpetrator or the victim.

Finally, the emphasis of person-related content refers to the reporting of the relevant situations and events in the form of personal interviews, with the victims or the perpetrators as the main body. Similarly, it was also divided into “victim-centered”, “neutrality” and “perpetrator-centered”.

(7) Attribution of case responsibilities

In this study, the case responsibility attribution was divided into the perpetrator responsibility attribution framework and the victim responsibility attribution framework, and “the perpetrator or the victim of the case who is more responsible” was set in the analysis of sexual crime reports. According to previous research (Young-Jun Son, Juhyun Hong, 2018), there are two kinds of analysis variables. First, the responsibility attribution of the perpetrator and the victim that has been specifically identified in the sexual crime reports. Second, the responsibility of the perpetrator or the victim has not been directly indicated. For example, “The female employee was drunk in the early morning and walked alone in the street in thin clothes”. Although the direct cause of sexual crimes is not mentioned, one of the reasons for these reported sexual crimes is “a victim who is drunk and has thin clothes”, which is a way to lure

people into committing crimes, and the victim is partly responsible for the occurrence of the case. Based on the approach of antecedent research, the behavioral judgment of the victim or perpetrator of sexual crimes in context was followed, and who is to be blamed for the cause of the case was judged according to the attribution effect on a three-point scale, with 1 point for "not mentioned at all", 2 points for "mentioned to a certain extent" and 3 points for "specifically mentioned".

(8) Thematic frame and episodic frame

The research on news frames focusing on news composition forms was divided into the thematic frame and episodic frame (Dong-Hoon Lee,·Won-Yong Kim,2012). Based on the previous research, the focus of "What happened at what time and under what circumstances?" and "Who said what?" was on the report of a real list of cases, which was defined as the episodic frame of sexual crime news. Instead, "Why did this happen?" "What are the main points of contention?" "What is the solution?", is the thematic frame that attempts to comprehensively analyze the causes, impacts, and solutions. Besides, "episodic frame + thematic frame" was added because of the double frame, and other frames were set as "Other".

(9) Coding and reliability verification

To ensure the reliability verification of the analysis items, the reliability verification was carried out with the help of Professor Li Deshun, associate professor of communication at Huaiyin Normal University in China. The encoding was tested based on approximately 5% of the extracted 600 articles and a final random sampling of 30 articles.

To analyze the content objectively and ensure the reliability between codes, in this study, the Holsti(1969) method commonly used in the content analysis was used to test the reliability

of materials by percentage for mutual consistency. The inherent consistent reliability of the sequence problems was tested by the Cronbach α method.

The results showed that the reliability of each type of nominal problem was above 0.9, which verified the content reliability of the data used in this study. The sequence question was Cronbach's alpha = 0.658, which validated the reliability of the content of the data used in this study.

6. Research Results

(1) Quantity of news reports about sexual crimes

Table 1. Number of news reports about sexual crimes in People's Daily Online

Years	Number of news reports
In 2004	2 (0.0%)
In 2005	9 (0.1%)
In 2006	3 (0.0%)
In 2007	10 (0.1%)
In 2008	34 (0.2%)
In 2009	7 (0.1%)
In 2010	6 (0.0%)
In 2011	1,200 (8.6%)
In 2012	2,423 (17.4%)
In 2013	5,027 (36.2%)
In 2014	2,745 (19.8%)
In 2015	1,186 (8.5%)
In 2016	463 (3.3%)
In 2017	285 (2.1%)
In 2018	231 (1.7%)
In 2019	266 (1.9%)
Total	13,897 (100.0%)

Note: (unit: piece, (%))

Table 1 shows the number of news related to sexual crimes each year. From 2004 to 2019, there were a total of 3,897 pieces of news on sexual crimes. On the whole, there were relatively few related reports from 2004 to 2010, and the first news of sexual crimes appeared on December 3, 2004. As of April 21, 2009, a total of 59 news articles were all related to Japan,

with a slight increase in 2008 due to the occurrence of juvenile sexual crimes by the US military in Japan, accounting for 18 out of 34 news articles. From 2004 to 2010, the total number of news reports related to all sexual crimes was 71 (0.5%). In the early days, most of the news retrieval results about sexual violence articles in China were concentrated in Japan, especially the reports about comfort women, the Second World War, and other historical events. From 2011 to 2015, the number of relevant news articles exceeded 1,000 each year, especially in 2011, 2012, 2013, and 2014, which was significantly different from the number of original full-size specimens, because irrelevant news in the data editing process and the situation of repeating the same news in one day were excluded. Since then, from 2016 to 2019, news related to sexual crimes has once again shown a decreasing trend year by year.

(2) The place where the cases reported by the news about sexual crimes occurred

Table 2. Analysis results of places where cases of sexual crimes are reported by People's Daily Online

Place of crime	Frequency	Percentage (%)
1) Chinese mainland	242	40.3
2) Hong Kong, Macau and Taiwan, China	86	14.3
3) South Korea and Japan	68	11.3
4) Other Asian countries	94	15.7
5) The United States, Europe and Australia	84	14.0
6) Other countries (Africa, Latin America, etc.)	26	4.3
Total	600	100.0

Note: (unit: piece, (%))

According to Table 2, the results of frequency analysis on the places where sexual crimes are reported by People's Daily Online show that "Chinese mainland" had the largest number of reported sexual crimes with 242 cases (40.3%), followed by "other Asian countries" with 94 cases (15.7%). Although the number of news items in Chinese mainland was high on the whole,

news from other regions as a whole accounted for 60% (358 pieces). That is to say, news from other regions combined accounted for more than half. Thus, the People's Network reported more cases of sexual crimes in other regions than Chinese mainland.

(3) Forms of news reports related to sexual crimes

Table 3. Results of the analysis on the report form of the People's Network Online news on sexual crimes

Report form	Frequency	Percentage (%)
1) Text	421	70.2
2) text and pictures	148	24.7
3) Video	26	4.3
4) Video and text	4	0.7
5) Video, pictures, and text	1	0.2
Total	600	100.0

Note: (unit: piece, (%))

According to Table 3, the results of frequency analysis on the forms of news reports about sexual crimes in People's Daily Online show that the forms of news reports about sexual crimes are mainly "text". The forms of sexual crime news reports in People's Daily Online are more traditional statements.

(4) Information sources of news about sexual crimes

Table 4. Results of analysis on information sources of news on sexual crimes in People's Daily Online

Information source	Frequency	Percentage (%)
1) Government agencies and their employees	395	51.0
2) Non-governmental social organizations	44	5.7
3) Experts and scholars	83	10.7
4) Parties and their families	108	13.9
5) Witnesses and relevant information providers on the spot	47	6.1
6) Unclear information sources	54	7.0
7) Others	44	5.7
Total	775	100.0

Note: (unit: piece, (%))

According to Table 4, the results of frequency analysis of news sources of sexual crimes reported by People's Daily Online show that 395 (51%) news sources related to sexual crimes were "government agencies and their employees". On the whole, more than half of the news related to sexual crimes seek authoritative and reliable information sources such as "government agencies" and "experts and scholars". It thus can be judged that the sources of sexual crime-related news in China are generally obtained directly from the government and other highly trusted channels and the parties concerned.

8) Is there a celebrity in the news reports about sexual crimes

Table 5. Analysis results of whether the relevant persons in the news reported by People's Daily Online about sexual crime are celebrities

Celebrity or not	Frequency	Percentage (%)
1) Entertainers	67	11.2
2) Celebrity	33	5.5
3) Ordinary people	500	83.3
Total	600	100.0

Note: (unit: piece, (%))

According to Table 5, the results of the frequency analysis on whether the persons related to sexual crimes reported by People's Daily Online are celebrities show that the persons related to sexual crimes reported by People's Daily Online are at most 500 "ordinary people" (83.3%), followed by 67 "entertainers" (11.2%). Thus, in the reports of People's Daily Online on sexual crimes, the related persons are mainly ordinary people, not celebrities or entertainers.

9) Media attention on perpetrators/victims of sexual crimes-related news reports

In this study, the media attention of the perpetrators/victims of sexual crimes reported by

People's Daily Online was analyzed from three dimensions, that is, the emphasis on news topics, the disclosure of personal information and the related content of persons, to analyze the media emphasis of all parts of the news.

Table 6. Results of the analysis on the topics of news reports on sexual crimes in People's Daily Online

News topic	Frequency	Percentage (%)
1) Neutrality	74	12.3
2) Victim-centered	128	21.3
3) Perpetrator-centered	267	44.5
4) Other	131	21.8
Total	600	100.0

Note: (unit: piece, (%))

Table 7. Results of the disclosure of personal information of perpetrators/victims in news reports of sexual crimes by People's Daily Online

Disclosure of personal information	Mean	Frequency	Standard deviation	Average standard deviation
Disclosure of personal information of perpetrators	9.91	600	2.72	.11
Disclosure of personal information of victims	8.93	600	2.10	.09

Note: t= 7.805, df=599, p<.05*

Table 8. Analysis results of information disclosure index of perpetrators in sexual crimes news reports of People's Daily Online

Disclosure of the perpetrator's personal information	Frequency	Mean	Standard deviation
(1) Name	600	1.93	.88
(2) Age	600	1.52	.87
(3) Face	600	1.22	.60
(4) Address	600	1.25	.53
(5) Employer	600	1.64	.90
(6) Exposure to people around	600	1.15	.46
(7) Exposure to private life	600	1.21	.54

Note: n=600

Table 9. Analysis results of information disclosure index of victims in sexual crimes news reports of People's Daily Online

Disclosure of victim's personal information	Frequency	Mean	Standard deviation
(1) Name	600	1.26	.56
(2) Age	600	1.71	.91
(3) Face	600	1.06	.31
(4) Address	600	1.23	.52
(5) Employer	600	1.47	.83
(6) Exposure to people around	600	1.13	.45
(7) Exposure to private life	600	1.07	.30

Note: n=600

Table 10. Results of the analysis on the emphasis on relevant content of sexual crime reports in People's Daily Online

Media attention	Frequency	Percentage (%)
1) Neutrality	297	49.5
2) Victim-related content emphasis	94	15.7
3) Perpetrator-related content emphasis	209	34.8
Total	600	100.0

Note: (unit: piece, (%))

Table 6 shows that 44.5%(267 articles) of the media coverage on sexual crimes focused on "perpetrators", while 21.3%(128 articles) focused on "victims". There is a difference of about 2 times between the news topic centered on the perpetrator and the news topic centered on the victim. The results of the analysis showed that journalists paid more attention to and use topics related to the perpetrators to attract readers when editing news.

Secondly, the disclosure degree of personal information of perpetrators/victims of sexual crimes reports was analyzed. As a result, the perpetrators' personal information disclosure degree was 9.9, and the victims' personal information disclosure degree was 8.9, that is, the perpetrators' personal information disclosure degree was higher than the victims. According to

the t-test results of the perpetrator/victim personal information disclosure specimen, there was a statistically significant difference in the scores of the perpetrator and victim personal information disclosure. As a whole, the personal information of the perpetrator was more open than that of the victim. According to Tables 8 and 9, the results of the analysis of the information disclosure index showed that “name” was the most exposed when disclosing the personal information of the perpetrator. On the contrary, the “age” of the victim’s personal information was most exposed, which showed that the age of the victims was paid more attention to during the editing process.

Finally, the related contents of the persons in the sexual crimes report were analyzed. According to Table 10, 34.8% of sexual crime reports emphasized the perpetrator and 15.7% emphasized the victim. If the ratio of emphasis between the perpetrator and the victim was compared, the perpetrator was relatively high. But on the whole, neutral content accounted for 49.5%, almost half of the sexual crime news reports.

10) Attribution of responsibility between perpetrators and victims in news reports related to sexual crimes

Table 11. Analysis results of responsibility attribution to perpetrators in sexual crime news reports of People’s Daily Online

Attribution of responsibility to the perpetrator	Frequency	Percentage (%)
1）Not mentioned at all	277	46.2
2）Mentioned to a certain extent	157	26.2
3）Specifically mentioned	166	27.7
Total	600	100.0

Note: (unit: piece, (%))

Table 12. Analysis results of responsibility attribution to victims in sexual crime news reports of People's Daily Online

Attribution of responsibility to the victim	Frequency	Percentage (%)
1）Not mentioned at all	563	93.8
2）Mentioned to a certain extent	31	5.2
3）Specifically mentioned	6	1.0
Total	600	100.0

Note: (unit: piece, (%))

Tables 11 and 12 show the analysis results of the responsibility attribution of perpetrators and victims in the news reports of sexual crimes in People's Daily Online. First of all, 46.2% of the perpetrators were "not mentioned at all". On the contrary, 93.8% of the responsibility attribution to victims of sexual crime-related reports was "not mentioned at all". The overall results showed that more than half of the reported contents pointed to the responsibility of the perpetrators.

11) Thematic frame/episodic frame in news reports related to sexual crimes

Table 13. Results of the analysis on the frame of news reporting on sexual crimes by People's Daily Online

Frames	Frequency	Percentage (%)
1) Thematic frame	239	39.8
2) Episodic frame	315	52.5
3) Thematic frame + Episodic frame	42	7.0
4) Other frame	4	.7
Total	600	100.0

Note: (unit: piece, (%))

According to Table 13, according to the results of the analysis on the frame frequency of the news reports on sexual crimes by People's Daily Online, 239(39.8%) articles adopted thematic frames, 315(52.2%) adopted episodic frames, and 42(7%) articles adopted both

simultaneously. The episodic frame was more frequently used in sexual crime news.

7. Conclusions and Discussions

In the past, Asian countries generally showed an evasive attitude towards sexual crimes, not only because of the sensitivity of the focus issues but also because of the national cultural background. Nevertheless, people pay more attention to sexual crimes with the progress of society. However, reporting distorted crimes to satisfy readers' curiosity or exposing personal information and reports without permission will not only leave lifelong pain for the parties involved but also form a "sea of public opinion" that will affect legal trials. Such a series of erroneous media reports will lose their essential functions and significance. With the highly developed information and the rapid development of media, the science of media communication, whether in application or theory, must be comprehensively and systematically understood, and the correct method of news dissemination must be deeply grasped. Obviously, it is important and necessary to find out the fundamental and structural reasons for reporting and analyzing sexual crime. Therefore, the discussion and enlightenment of the results of this study are as follows.

Firstly, the differences in terms used in reporting sexual offenses on People's Daily Online. Although they are all related to sexual crime, the use of vocabulary varies according to the theme of news content due to the context in which the Chinese language is used. Crime reports in China mostly have the characteristics of legal language. In other words, words like "sexual assault" and "sexual crime" mostly use official Chinese legal vocabulary, and "rape" is a

general narrative expression. “Sexual violence” is translated directly by countries like Japan and South Korea and is often used in their reporting.

Second, the difference in the number of sexual crime reports on the People’s Daily Online. From December 3, 2004, to April 27, 2009, all keyword-based news was associated with Japan, and since then, reports in other regions have increased. From 2011 to 2015, there was a significant increase in the number of news related to sexual crime, while the proportion of reports from other regions was also increased. From 2015 to 2019, news related to keywords showed a trend of gradual decrease again. According to analysis, at this stage, the number of false news and story reports decreased significantly, while the number of normative news and official court news increased. Judging from the changes, crime has been strengthened and prevented from the national perspective, thus the sexual crime rate has been reduced. Thus, the statistical results reveal a link between national policy reforms and the media.

Thirdly, about the location, report form, information source, whether there are celebrities and other related issues of the sexual crimes reported by People’s Daily Online. On the location where the incidents were reported, nearly 80% of the cases related to early sexual crimes were reported outside of mainland China. But in recent years, the news in the Chinese mainland has increased significantly, indicating that China is actively addressing the problem of sexual crimes. In the form of reporting, People’s Daily Online, as an authoritative media, mainly reports with texts and does not publish photos. As for the sources of news reports, People’s Daily Online tends to adopt channels with high trust and credibility from governments and institutions, but it also indicates that the channels of news sources are relatively single. China is different from other countries in whether the people are famous or not. There are few reports

on famous people, and it prefers the reports of ordinary people.

Fourthly, the part about the public opinion concern of the perpetrators/victims in the news reports of sexual crimes in People’s Daily Online. The study found that when reporters edit news, they pay more attention to and make use of the related topics that focus on the perpetrators to attract readers and use more topics that focus on the perpetrators in their reports. However, judging from the title of the report, there are many phenomena of using words that arouse sympathy for the perpetrators. For the most part, the personal information of the name of the perpetrator is directly exposed, but the personal information of the victim is mainly disclosed at “age”. It can be concluded that the point of most concern for the victims in the process of editing news is age.

Fifthly, the attribution of the responsibilities of the perpetrator/victim in the report. The analysis shows that more than half of the people focus on the perpetrators rather than the victims. It avoids wrong reporting methods and does not find reasons from victims, thus avoiding the phenomenon of a distorted interpretation of the attribution of responsibility for sexual crimes.

Sixthly, the form of sexual crime reporting frame in People’s Daily Online. The episodic frame was widely used in early news, but in recent years, the thematic frames have increased greatly, while the episodic frame has been used less. In recent years, the reports are more about the causes of incidents, national policies, legal discussions, and public opinions on social issues to guide the occurrence of sexual crimes, and how to prevent and solve problems, instead of simply describing the process of sexual crimes and attracting readers’ attention. Some negative reports on sexual crimes by People’s Daily Online mainly show criticism of the perpetrators’ criminal behaviors. While criticizing, it emphasizes that the government is constantly

improving the relevant laws or interpreting the effective legal policies already implemented to win the trust of the people.

As reporting on sexual crimes has often become the focus of social attention, the leading role of the media is very important in the new media era to build the reporting system and framework of sexual violence crimes, guide the awareness and public opinion of the people, and improve the vigilance of the society on sexual crimes. The media can not only play the right role of social organization to provide guidance and support for the victims' social life and mental health but also give them the right cognition and prevention functions for sexual crimes. Therefore, how to correctly report the news of sex crimes should be the topic that journalists constantly pay attention to.

REFERENCES

Ru Xi Jia (2019). A Preliminary study on the news reporting strategy of girl sexual assault. The Chinese newspaper, 480(23),105-106.

Hong, J. H. (2018). Network analysis and frame analysis on the sensationalism of news coverage according to the influence of news production environment: Based on the# metoo movement of celebrity. The Journal of the Korea Contents Association, 18(7), 103-119.

Ahn Kwang-sik (1998). Television crime Report and public opinion ethics. KWANHUN JOURNAL, 12(69), 224-237.

Jungah Ahn (2017). Framing Terror in the News Reports of CCTV, CNN, and KBS, Asia-pacific Journal of Multimedia Services Convergent with Art, Humanities, and Sociology, Vol.7, No.8, August (2017),201-210.

Xiao Wei (2015). On the Framework of News - The structure and the structure of the subject of communication. China Renmin University Press.

Wang Lingning (2009). A review of research status of domestic news framework. <Academic Journal of Zhongzhou>, 2009(06), 253-255.

Feng Qunxing (2014). Conflict of rights and media anomie in Sex crime News Reports -- Based on the analysis of "Li Case" related reports. News of the world, 2014(5), 257-259.

Yong-kuk Chung (2012). Media that use sex crimes as a "topic" are losing trust - three key points to examine in reporting sex crimes. Newspapers and radio, 503, 24-27.

Lippmann, W. (1922). Public Opinion. Chung Hun Lee (translation) (2012). < Public opinion >.

Seoul: Kachi Publishing.

Ju-Hyun Hon (2016). An Analysis of Diffusion of Main Information and Peripheral Information: Focusing on Visibility and Connectivity of Word based on Network Analysis. JOURNAL OF THE KOREA CONTENTS ASSOCIATION,16(3), 269-287.

Seon-A Moon, Bong-Geun Kim, Jin-Suk Kang. (2015).A Grounded-Theoretical Analysis on Report Attitude of Sexual Violence Crime: Focused on Qualitative Depth Interview with Journalists. Korean Journal of Broadcasting and Telecommunication Studies,29(6),37-66.

Greer, C. (2007). News media, victims and crime. 21-49. in Victims, Crime and Society, Pamela D., Peter F. and Greer. C. SAGE.

Gilchrist, K. (2010). “News worthy” victms? Exploring differences in Canadian local press coverage of missing/murdered Aboriginal and White women.

Sae-Eun Kim, Namhee Hong. (2019). News Media and the #Metoo Movement: Problematising the Practices of Korean Journalism Organisations. Media, Gender & Culture,34(1),39-88.

Worthington, N. (2008). Local TV news framing of acquaintance rape on campus. Feminist Media Studies, 8(1), 1-16.

Yisook Choi, Eunjin Kim. (2019). Whose voices were heard?: An analysis of TV news interviews about #Metoo.Media, Gender & Culture, 34(2),147-189.

Jung Hye Yang. (2010). News Representation of Crime: the Case of Sexual Violence against Children. Journal of Communication Science, 10(2), 343-379.

Price, V & Tewksbury, D & Powers, E (1997). Switching trains of thought: The impact of news frames on readers’ cognitive responses, Communication Research, 24, 1997, 481-506.

Z. Pan & G. M. Kosicki (1993). ‘Framing analysis: An approach to news discoures,’ Political

Communication, Vol.10, No.1, pp.55-75.1993.

Young Jun Son, Juhyun Hong. (2018).TV News Coverage of Sexual offences: Focusing on Attention of News, Disclosure of Personal Information, Incident Attributes. Korean Journal of Broadcasting and Telecommunication Studies, 32(3), 31-68.

Semetko, H. A., & Valkenburg, P. M. (2000). Framing European politics: A content analysis of press and television news. Journal of Communication, 50(2), 93-109.

Lu, Jian Ye, Chung, Yongkuk. (2019).A Study on the Effects of News Frame and Issue Involvement on Audience Responses, Issue Perceptions, and Responsibility Attributions. THE JOURNAL OF SOCIAL SCIENCE, 26(4),117-142.

Jung-Gyo Lee, Young-Nam Seo, Su-Jin Choi. (2009). Exploring Rape Reports in Newspapers - A Longitudinal Analysis of Four Korean News Dailies between 1990 and 2007.Korean Journal of Communication & Information, (45), 425-462.
