# Supplementary material for: Reports on sexual violence published in an online Chinese newspaper: A new frame research
Source: PLoS One. 2024 May 10;19(5):e0299578. doi: 10.1371/journal.pone.0299578 (PMC11086924; doi:10.1371/journal.pone.0299578)
Supplement: Attachment — Submitted filename: TdV_PlosOne Review_August2023.docx [file pone.0299578.s002.docx]

**Reviewer report: 31 August 2023**

Thank you for allowing me to review this manuscript. See my comments below.

- In my opinion, the claims of the paper, i.e., how sexual crimes are reported in the media and the role of conveying this information to the larger society, is an important area of research, and should be published.
- In terms of the literature: On page 4, second paragraph, third line, highlighted in green, the author refers to “*According to the first research in this field* …” but the reviewer could find no evidence to support this statement. The author should make this research referred to, known to the reader or not refer to it as such.
- The data and analyses support the claims made by the author.
- The supporting data align with the research questions.
- The paper in its current form is not suitable for publication, but with editing and changes suggested, I think the study itself shows sufficient potential that the authors should revise and resubmit. For example, the paragraph highlighted in yellow in the abstract needs to be reworked, because it was confusing to the reviewer. The first sentence in the introduction, *“ … the actress …*” is missing a name. Should the second last phrase, highlighted in yellow at the bottom of page 2, not read “*social justice”*? On page 7, the last sentence under (3), that reads *“ … they will be highly concerned by the media*” Is not clear to the reviewer. The author may want to revise this sentence or provide a clearer description for the reader to understand. The last sentence, highlighted in yellow, under “3. Research Questions” states: “This study will analyse …”. Was the study not already conducted? If yes, then it should state: “This study analysed …”. Can the author have a look at this?

On page 12, under “4 Material Selection”, it would look better if the author presented the information in a table format. The definition of terms are in the body of the paper. My suggestion would be to have this earlier in the paper, then the researcher understands the definition when it comes up later in the paper/

- I think this paper is well organised and accessible, even to non-specialists. Is the manuscript well organized and written clearly enough to be accessible to non-specialists?

Sincerely

Dr Tania de Villiers
