# Supplementary material for: Reports on sexual violence published in an online Chinese newspaper: A new frame research
Source: PLoS One. 2024 May 10;19(5):e0299578. doi: 10.1371/journal.pone.0299578 (PMC11086924; doi:10.1371/journal.pone.0299578)
Supplement: Attachment — Submitted filename: reviewer#2.docx [file pone.0299578.s004.docx]

**Dear Reviewer, 2**

First, I would like to express my sincere gratitude for your valuable comments during the review of our manuscript. The issues you raised are crucial to our research, and this feedback has played a key role in improving the quality and depth of our article.

Based on your suggestions, we have made detailed revisions to the structure and presentation of the full text. We have ensured that all key points and statements are well supported by the literature, while providing clearer presentation and accurate references to enhance the persuasive and scientific nature of the article. We have highlighted the review comments in green throughout the text.

1） **Title**: I propose the following essay “Reports on sexual violence published in an online Chinese newspaper: a new frame research”

We changed the title to “Reports on sexual violence published in an online Chinese newspaper: a new frame research”

2） **Abstract**:

- It does not present information related to the "materials and method" section.
- Review the wording of the excerpt "People's Daily Online, a representative online media in China, was taken as the research object, to analyze the sexual crime reports in the past 15 years.”, in order to make the objective clear to the reader of the article.
- Pay attention to the time frame mentioned, which is not the same as mentioned in the text of the manuscript, where the authors report 20 years.

We have made changes to these three issues. We have expanded the Materials and Methods section. This includes 3.1 Research design, 3.2 Material selection.

3） **Introduction**: Does not present the purpose of the manuscript at the end of the section. As a suggested objective, I propose: To describe the characteristics of the news about sexual violence published in the People's Daily Online.

We have included the purpose and research questions of this study in the last paragraph of the introduction section, which is highlighted in green. “The purpose of this study is to provide an in-depth analysis of the coverage of sex crimes in recent years in China's main online media, the People's Daily. The study examines various aspects of this coverage, including the volume of sex crime news, the nature of the content, the areas of focus, the attribution of responsibility and the framing techniques used. This in-depth investigation aims to shed light on the Chinese media's attitudes and approaches to reporting sex crimes, as well as the impact of such reporting on societal attitudes and values.

In the conclusion and discussion section, this study aims to highlight the important role that media coverage plays in raising social awareness of sexual crimes, shaping public ideology and upholding social justice. By providing a comprehensive and in-depth examination of the coverage of sexual crimes in China, this study offers valuable insights and recommendations that are relevant to future considerations in terms of social health, psychological awareness, media communication and policy development.“

# 4）  Theoretical Background:

- The title level of subsections is not presented according to publication standards.

We have updated the subsection headings in Chapter 2.

**2. Conceptual Definition and Related Literature**

2.1 Conceptual Definition

2.2 Related Literature

(1) Literature review of sexual crime reports
(2) Problems with sex crimes
(3) Obstacles to coverage
(4) The tool “frame” of news report analysis

- Note that, in some paragraphs, there are excerpts with wording that resemble the objectives of the study.

We have reorganised the section.

- Review the last paragraph of this section, as its content has multiple objectives, which is not appropriate for a scientific article.

We have deleted parts and kept only the last paragraph. Through the discussion of the research, this paper deeply narrates the news statement theory, summarizes the attribution theory which serves as the theoretical basis of responsibility attribution, and then focuses on the responsibility attribution to describe the frame theory.

- Research Questions: This is not a section of the manuscript in accordance with the journal's guidelines. I suggest including the content presented in item 3 in the introduction, making the necessary adjustments in the wording.

We have removed the small section on research questions and included it in the introduction.

“The purpose of this study is to provide an in-depth analysis of the coverage of sex crimes in recent years in China's main online media, the People's Daily. The study examines various aspects of this coverage, including the volume of sex crime news, the nature of the content, the areas of focus, the attribution of responsibility and the framing techniques used. This in-depth investigation aims to shed light on the Chinese media's attitudes and approaches to reporting sex crimes, as well as the impact of such reporting on societal attitudes and values.

In order to better understand these dynamics, this study asks several key research questions:

1) What are the characteristics and trends in reporting of sexual offences on the People's Network?
2) What are the changes in the locations of sex crimes reported on People's Daily Online?
3) What are the trends in the form of sexual crime reporting on People's Daily Online?
4) What are the trends in the news sources of People's Daily Online's sex crime reports?
5) How often does People's Daily Online‘s report on celebrity sex crimes?
6) Is there a difference in media attention between perpetrators and victims in the People's Daily Online sex crime stories?
7) How does the People's Daily Online allocate responsibility between perpetrators and victims?
8) What is the relationship between thematic and situational reporting in People's Daily Online sex crime stories?

- Regarding the guiding questions of the research, I recommend the presentation of only one guiding question, which encompasses the central idea. For example: what are the characteristics of the news about sexual violence published in People's Daily Online?

9) Changes have been made to the first research question in the introduction: “What are the characteristics and trends in reporting of sexual offences on the People's Network?”

**5) Methodology**: The title of this section, according to the rules of the Journal is “Materials and Methods”. Still, it is necessary to critically review the text considering the recommendations of the COREQ checklist and the journal's rules regarding the structure of this section.

- The content presented in this section does not allow the replicability of the study. The methodological path developed by the authors is not clear.
- I recommend describing the data collection instrument applied to the reports.
- What is the type of study?
- What was the method adopted for data collection? It is necessary to cite the reference used as the basis for the methodological route.
- What version of the SPSS 19.0 program was used?
- Justify the change in the time frame considered the survey of publications. Initially the proposal was 20 years, but “from January 1, 1997, when People’s Daily Online was established, to December 31, 2019”, totals 22 years and 11 months. Next, the authors say that the cut was from October 10, 2004, to December 31, 2019”, which totals 15 years and 2 months. The reader is confused and this information conflicts with that presented in the abstract.
- When did data collection take place? who performed it? what was the instrument adopted for data collection?
- Since Python is a programming language, the text needs to be more specific about how this language was used. Through a search engine? Crowler? Which network did Python access? Python is a programming language, but was the feature adopted for research purposes? Justify the use of this language in the research.

For this section, we have reorganised the structure of the article, revised 3.1 Research design and 3.2 Material selection, and added an elaboration of the data collection method and the necessary justification.

In this study we explored in depth the issue of reporting of sexual offences over the 15 years since the People's Network was established. The research process was carried out in four stages. First, in the data import phase, we used the Python programming language to run a web crawler (crawler) to automate the collection of sex crime-related news reports from the People's Daily Online (http://search.people.cn).Python is known for its powerful libraries and frameworks such as Beautiful Soup and Scrapy, which are widely used for web crawler development, and these tools can efficiently parse and extract web content.

Specifically, our Python crawler targeted the news archive section of People's Daily. By setting specific keywords such as 'sex crime', 'rape', 'sexual violence', and 'sexual assault', the crawler automatically searched for keywords related to these terms. The crawler automatically searched for news articles related to these keywords and extracted data such as publication date, news content, and links. This method is an efficient and accurate way to filter relevant stories from a large amount of data and provides reliable raw data for this study. The collected news release dates, contents, and links were also organised in an orderly manner in Excel. The second stage of data cleaning involved the precise screening of all the collected reports to ensure that only those directly related to sexual offences were included. In the third stage, I sampled the collected reports using a systematic sampling method. Finally, in the stage of statistical analysis and interpretation of information, IBM SPSS Statistics 19 software was used to perform frequency analysis of relevant content.

The time span of this study is from 1 January 1997, the date of the establishment of the People's Network, to 31 December 2019, the date of its establishment. However, the actual time period analysed was determined based on the availability of relevant data, i.e. from 10 October 2004 for "sexual offences", "rape", "sexual violence" and "sexual assault", and from 10 October 2004 for "sexual offences", "rape", "sexual violence" and "sexual assault" to 31 December 2019 for "sexual offences", "rape", "sexual violence" and "sexual assault". "Sexual assault" from 10 October 2004 to 31 December 2019, when the keywords "sexual offence", "rape", "sexual violence" and "sexual assault" first appear in reports. This timeframe provides a comprehensive view of the evolution of coverage of sexual offences over time, capturing dynamic changes and trends in media coverage.

- What were the inclusion and exclusion criteria adopted for the selection of news?
- Explain the adoption of 5% of publications for coding
- The authors present “Operational definition and analysis” as a new section of the manuscript, however, its content is part of the “Materials and Methods” section, so it must be presented as a second level of subtitle. :

As shown in Table 1, a keyword search was conducted using Python and a total of 80,385 stories were captured. Based on the results, 3637 stories related to "sexual crimes", 52,435 stories related to "rape", 3228 stories related to "sexual violence", 21085 stories related to "sexual assault", a total of 80385 stories. "Sexual assault" stories 21085, total 80385 stories. First, all the news items related to the four keywords were sorted in chronological order. Then the samples were screened according to the following requirements:

1) delete the news articles that repeated each other; 2) delete the news articles that repeated each other; 3) delete the news articles that repeated each other.

2) Delete "comfort women", "World War II" and historical content.

3) Delete news that is not related to sex crimes, such as "OO sex crimes" (e.g., violent sex crimes, group sex crimes, etc.).

4) Domestic violence, resource-based sex trafficking and other related issues are not included in this study.

5) Reporting of people with previous convictions for sexual offences for committing other crimes.

6) Misreporting (e.g. direct statements in the news, non-existent cases, etc.).

7) Cases where the same topic or news title is different but has exactly the same content on the same day.

8) Exclusion of content not related to sexual offences (e.g. film introductions, novel recommendations), etc.

Based on previous research, the research sample selected for this study is the series of news about sexual crimes, i.e., the occurrence of the case, the initiation of the police investigation, the arrest, the detention until the indictment, the indictment until the verdict of the trial, and the reporting and investigation of the case by the investigating body before the initiation of the investigation (Roh et al., 1996). In addition, the study sample included news of cases of forced prostitution, news of clandestine filming, and published news of sexual violence, as well as comprehensive reports and comments on sexual violence. The data were cleaned according to the definitions and samples that did not meet the requirements of the study were excluded. In the end, 13,897 samples were collected.

A systematic sampling method was used to select 5% of 20 articles at regular intervals, resulting in a total of 694 news articles on sexual violence. By 23 June 2012, 87 news articles in the test sample had expired and only 7 articles were available for submission. Thus, the final number of valid news articles for analysis was 600, with 5% of the sample coded by the coder and measured for intercoder reliability testing.

- As numbered sub-items, the authors present the variables considered in the study, that is, what was extracted from the data. This information should be presented in a second subheading level called “Data collection”.

Changes have been made to the content of the subheadings in the relevant sections.

3.2 Material selection

- Pay attention that, when presenting the variables (the quantity of news reports, Place of crime, the characteristics of sexual crime reports, The information source of a report, who is the persons in the news, the focus of media attention, Attribution of case responsibilities and frames focusing), sometimes the authors present data, which is not appropriate for the “Methods” section, as well as presenting information regarding the analytical process.

Relevant content has been integrated with 4.Research Results

- From subitem 9 ("(9) Coding and reliability verification"), the authors present contents referring to a second level of subtitle of the section "Materials and Methods" called "Data analysis”.

Added to 3.2 Material Selection

# 6) Conclusions and Discussions:

- According to the journal's rules, the section to be presented after the results is the “Discussion” section, which can be presented together with the results. However, “Conclusions” is another section, which cannot be presented together with the “Discussion” section”.
- The text in this section lacks a broad critical review, as the content consists of the authors' interpretations of the results, without any inference from the authors or dialogues with the findings of other research. This note is evident when observing that no reference was cited in this section.
- I emphasize that the text presented does not reach the depth required for an analysis, as proposed by the authors.

7) References:

- The list of references does not follow the format required by the journal.
- I strongly recommend updating most of the references used, since out of a total of 22 references, 16 are publications older than 5 years.

6) and 7) We have reworked the conclusion and discussion sections of the paper and added relevant literature.

This study's in-depth analysis of the coverage of sexual offences in the People's Daily Online between 2004 and 2019 reveals its multifaceted characteristics and trends. First, fluctuations in the number of reports reflect shifts in public attention and adjustments in the media's strategies for covering sex crimes. In particular, the significant increase in the number of reports between 2011 and 2015 may be related to increased public awareness and media attention on the issue of sexual offences. The trend of fake news and related reports gradually decreases and the number of normative and court news increases between 2015 and 2019. In terms of location, the coverage is not only concentrated in mainland China, but also covers a wide range of other regions, showing the global perspective of the media. In terms of form of reporting, the preference for traditional texts was largely due to the careful handling of sensitive issues and the protection of victims' privacy.

In terms of news sources, the preference for government agencies and expert sources shows the importance attached to authority and reliability. In terms of coverage of people, stories of ordinary people dominated, but more attention was given to perpetrators than to victims, which may have influenced public perceptions and attitudes. In terms of disclosure of personal information, perpetrators were more prominent, reflecting the attention paid to the identity of perpetrators in the coverage. In terms of the nature of the content of the stories, although more attention was given to the perpetrator, neutral stories accounted for almost half of the coverage, demonstrating the balance and objectivity of the People's Daily Network in its coverage of sexual offences.

In our analysis of the People's Daily's coverage of sex crimes, we identified a number of key features and trends, but these findings also revealed areas for critical scrutiny. First, while fluctuations in the number of stories reflect changes in public attention, the excessive focus of stories on perpetrators may have led to the neglect of victims' experiences. This bias may not only lead to secondary victimisation but may also send the wrong message to society about ignoring the encounters and feelings of victims. As Benedict (1993) points out, such reporting practices can reinforce public stereotypes and misconceptions about victims of sexual crime.

Furthermore, although coverage shows geographical diversity, it is important to critically consider whether this diversity truly reflects the complexity of the global crime problem. Research by Jewkes (2015) suggests that the media may tend to cover sexual crime in certain regions while ignoring others, which can lead to a one-sided understanding of the global crime problem. At the same time, an over-reliance on government and expert sources of information can limit the perspective and depth of reporting. Schlesinger and Tumber (1994) highlight that this contributes significantly to a one-sided reporting of sexual crime issues, ignoring other important social and cultural factors.

The choice of reporting framework is also a key issue. The use of an episodic framing format, while enhancing the appeal of the story, can also simplify the complexity of the sex crime issue. Entman (1993) points out that this can lead to a one-sided understanding of the sex crime issue among the public, ignoring the root causes of the problem and the social context. Furthermore, while the protection of victim information in reporting is commendable, this practice needs to be balanced against the comprehensiveness and transparency of reporting. Over-protection of victim information can lead to a lack of public understanding of the full picture of sexual offending.

Key areas for future research include an in-depth exploration of the ways in which sexual offences are reported and focused on in different geographical and cultural contexts, in order to provide a more comprehensive global perspective. There is also a need to analyse different news sources, including governments, non-specialist victims, social groups and public opinion, to ensure comprehensive coverage and multiple perspectives. At the same time, the project will focus on the impact of the People's Daily's coverage of sexual crimes on public perceptions and attitudes, particularly at a time when social media and digital technologies are becoming increasingly important. Exploring how to innovate reporting frameworks and methods to improve the quality of reporting and public engagement is also an important direction for future research. Finally, further research on journalistic ethics and responsibility, particularly in balancing truthfulness and comprehensiveness of reporting with respect and protection of victims, will be key to improving the quality of media coverage and social impact.

In conclusion, although the People's Daily has demonstrated multiple aspects in its coverage of sexual crimes, there is still room for critical review and improvement in terms of public impact, reporting strategies and journalistic ethics. In future media practice, while striving for balance, depth and diversity in coverage, there is a need to strengthen commitment to social responsibility and journalistic ethics in order to improve the quality of coverage and social impact.

We value every comment from our reviewers and your professional advice is invaluable to us. We look forward to your further review and feedback on the revised manuscript and hope that our efforts will meet your expectations and that we will be able to share our findings with the wider academic community.

Thank you again for your time and professional guidance. We look forward to receiving your valuable comments.
