# Supplementary material for: Reports on sexual violence published in an online Chinese newspaper: A new frame research
Source: PLoS One. 2024 May 10;19(5):e0299578. doi: 10.1371/journal.pone.0299578 (PMC11086924; doi:10.1371/journal.pone.0299578)
Supplement: Attachment — Submitted filename: reviewer R2#2.docx [file pone.0299578.s005.docx]

**Dear Reviewer, 2**

Reviewer #2: All reviewer queries have been addressed adequately. Ensure no dual publication. The manuscript was presented in an intelligible fashion.

Reply: Your comments have been invaluable in guiding our revisions and ensuring the clarity and integrity of our work.

Thank you again for your constructive feedback and guidance throughout the review process. We believe that your suggestions have significantly improved the quality of our manuscript.
